# Clinical utility of the GAD-7 for detecting generalized anxiety in Quechua indigenous people

**DOI:** 10.3389/fpsyt.2025.1565895

**Published:** 2025-05-30

**Authors:** Nicole Caldichoury, César Quispe-Ayala, Juan-Carlos Coronado, Luis Mario Castellanos-Alvarenga, David Salazar, Breiner Morales-Asencio, Daniela Ripoll-Córdoba, Raúl Quincho-Apumayta, Juan Cárdenas-Valverde, Loida Camargo, Karen Alcos-Flores, Eloy Layme-Condori, Rosa Villalba-Arbañil, Cesar Castellanos, Pascual A. Gargiulo, Indalecio Quispe-Rodríguez, Elsa Muñoz-Romero, Alberto Rivelino Patiño-Rivera, Irina Flores-Poma, Jorge Herrera-Pino, Norman López

**Affiliations:** ^1^ Departamento de Ciencias Sociales, Universidad de Los Lagos, Osorno, Chile; ^2^ Universidad Nacional de Huancavelica, Huancavelica, Peru; ^3^ Departamento de Procesos Terapéuticos, Facultad de Ciencias de la Salud, Universidad Católica de Temuco, Temuco, Chile; ^4^ Escuela de Psicología, Facultad de Ciencias Sociales y Comunicaciones, Universidad Santo Tomás, Temuco, Chile; ^5^ Universidad Nacional Daniel Alcides Carrión, Cerro de Pasco, Peru; ^6^ Consorcio Latinoamericano de Investigación (CLATI), Temuco, Chile; ^7^ Universidad Nacional Autónoma Altoandina de Tarma, Tarma, Peru; ^8^ Facultad de Medicina, Departamento Médico, Universidad de Cartagena. Grupo de investigación Neurociencia y Salud Global, Cartagena de Indias, Colombia; ^9^ Universidad César Vallejo, Lima, Peru; ^10^ Instituto dominicano para el Estudio de la Salud Integral y la Psicología Aplicada (IDESIP), Santo Domingo, Dominican Republic; ^11^ Laboratorio de Neurociencias y Psicología Experimental. Área de Farmacología, Facultad de Ciencias Médicas, Universidad Nacional de Cuyo, Mendoza, Argentina; ^12^ Universidad Nacional de San Cristóbal Huamanga, Ayacucho, Peru; ^13^ Facultad de Educación, Universidad Nacional Intercultural de la Selva Central Juan Santos Atahualpa, La Merced, Chanchamayo, Peru; ^14^ College of Medicine, Florida Internacional University, Miami, FL, United States; ^15^ Escuela de Kinesiología, Facultad de Salud, Universidad Santo Tomás, Temuco, Chile; ^16^ Departamento de Ciencias Sociales, Universidad de La Costa, Barranquilla, Colombia

**Keywords:** clinical utility, case-control study, GAD-7 test, rural indigenous, Quechua

## Abstract

The detection of Generalized Anxiety Disorder (GAD) in indigenous populations poses a challenge due to their holistic approach to health, which significantly contrasts with the Western biomedical model. Moreover, conventional assessment tools often overlook cultural particularities, compromising their effectiveness in these contexts.

**Objective:**

Therefore, this study aimed to analyze the diagnostic accuracy of the GAD-7 in the Quechua indigenous population of the Peruvian Andes.

**Method:**

To address this issue, we conducted a case-control study to evaluate the clinical accuracy of the Generalized Anxiety Disorder Test (GAD-7) in rural Quechua communities of the Peruvian Andes. We included 147 GAD patients and 322 controls. The study involved four stages: cultural adaptation of the GAD-7, door-to-door evaluation, blind psychiatric and neuropsychological assessments, and application of the Quechua GAD-7. The adaptation used the Delphi method, focus groups, and bilingual judges. Factor analyses, reliability assessments, and diagnostic utility evaluations were performed.

**Results:**

The Quechua GAD-7 showed high content validity (Aiken’s V > 0.85), strong internal consistency (α = 0.912, ω = 0.85), and an area under the curve of 0.93. With a cutoff score of 11, it achieved 91.3% sensitivity and 86.1% specificity.

**Conclusions:**

This is the first study to validate a Western test for GAD in indigenous populations.

## 1 Introduction

Generalized anxiety disorder (GAD) is a mental health condition characterized by excessive and uncontrollable worry (1). Its etiology is multifactorial, but chronic stress has been shown to predispose individuals to its development and amplify the associated emotional and physiological burden (2–4). Currently, GAD is one of the most challenging problems worldwide (5–7); Due to the complexity of GAD symptoms and their interaction with daily life, affected individuals often experience somatic and cognitive problems, such as motor restlessness, muscle tension, sleep disturbances, difficulty concentrating, fatigue, and irritability (8). Additionally, it often coexists with other psychiatric disorders, such as bipolar disorder, depression, and substance use (9–14). In some cases, GAD can even increase the risk of suicide, significantly impacting occupational and functional performance (15–17).

In Peru, the COVID-19 pandemic exacerbated the burden of anxiety, with rates ranging between 13.1% and 41.8% during this period (18–23); This phenomenon is well documented in the general population (24–31). However, knowledge about GAD in rural Indigenous communities, both globally and in Peru, remains limited (32, 33). In this regard, available evidence suggests that Indigenous populations face significant barriers to accessing appropriate medical and mental health services (34–38). This is due to factors such as geographical barriers (37), cultural differences, the lack of preparation among healthcare professionals, and economic aspects (39–41). Additionally, the symptoms of GAD often overlap with other mental disorders, complicating the diagnostic process (7). Finally, there are few validated clinical instruments available to detect anxiety symptoms in culturally diverse populations (42–46). This combination of factors hinders the early detection and treatment of GAD (47, 48). This underscores the need to develop and validate useful instruments for rural and indigenous populations in the country.

The GAD-7 is a self-assessment test, developed by Spitzer et al. in 2006 (49), to assess symptoms of generalized anxiety over the past two weeks. It has been widely used around the world because of its high sensitivity, simplicity, and ease of administration. The test consists of seven items that are statements about worry or somatic symptoms, rated on a four-point Likert scale. Factor analyses have shown that the GAD-7 has adequate psychometric indicators, explaining between 60% and 70% of the variance, with adequate fit indices (RMSEA = 0.080; CFI = 0.995; SRMR = 0.053) and high internal consistency, with a Cronbach’s alpha of 0.920, indicating high reliability for the detection of GAD symptoms, as demonstrated in various studies (50–55).

However, global evidence shows that most studies analyzing the statistical or clinical indicators of the GAD-7 have been conducted primarily in Western contexts and urban communities (50, 56–60); with some studies conducted in rural settings (61, 62). To our knowledge, the clinical utility of the GAD-7 has not been evaluated in indigenous communities living in culturally indigenous settings, such as rural contexts. Evaluating the diagnostic utility of the GAD-7 in a sample of Indigenous adults is critical to ensure that the test accurately captures the specific cultural manifestations of anxiety in this population. Since expressions of anxiety can vary significantly between urban and Indigenous contexts, validation of the GAD-7 in this setting will ensure that the language is understandable, the relevant symptoms are appropriately measured, and the scoring norms reflect the cultural realities of Indigenous populations. This is essential for improving diagnostic accuracy, ensuring the clinical relevance of the test, and designing mental health interventions that are culturally sensitive and effective. Therefore, the aim of this study was to analyze the diagnostic accuracy of the GAD-7 in the Quechua Andean Indigenous population of Peru.

## 2 Materials and method

Cross-sectional case-control study with blinded diagnostic assessment to analyze the diagnostic accuracy of a clinical scale for detecting generalized anxiety in a non-probabilistic sample of 147 patients with a medical diagnosis of GAD and 322 healthy controls; all participants were middle-aged adults, bilingual rural Quechua Indigenous individuals from the Peruvian highlands. Measurements were conducted between 2023 and 2024.

The sampling was carried out in two successive phases. First, through purposive door-to-door community sampling, bilingual Quechua Indigenous adults from rural areas of the Peruvian Andes were identified. Subsequently, targeted clinical sampling was applied, which allowed the selection of participants based on a blinded medical diagnosis of GAD or its absence. This non-probabilistic, clinically contrasted sampling strategy is common in diagnostic accuracy studies, particularly in contexts where there are limitations in clinical records and access to health services is limited(63). The combination of both phases allowed for the construction of a balanced and culturally relevant sample to evaluate the diagnostic accuracy of the GAD-7 in this context.

### 2.1 Procedure

**Figure 1:**
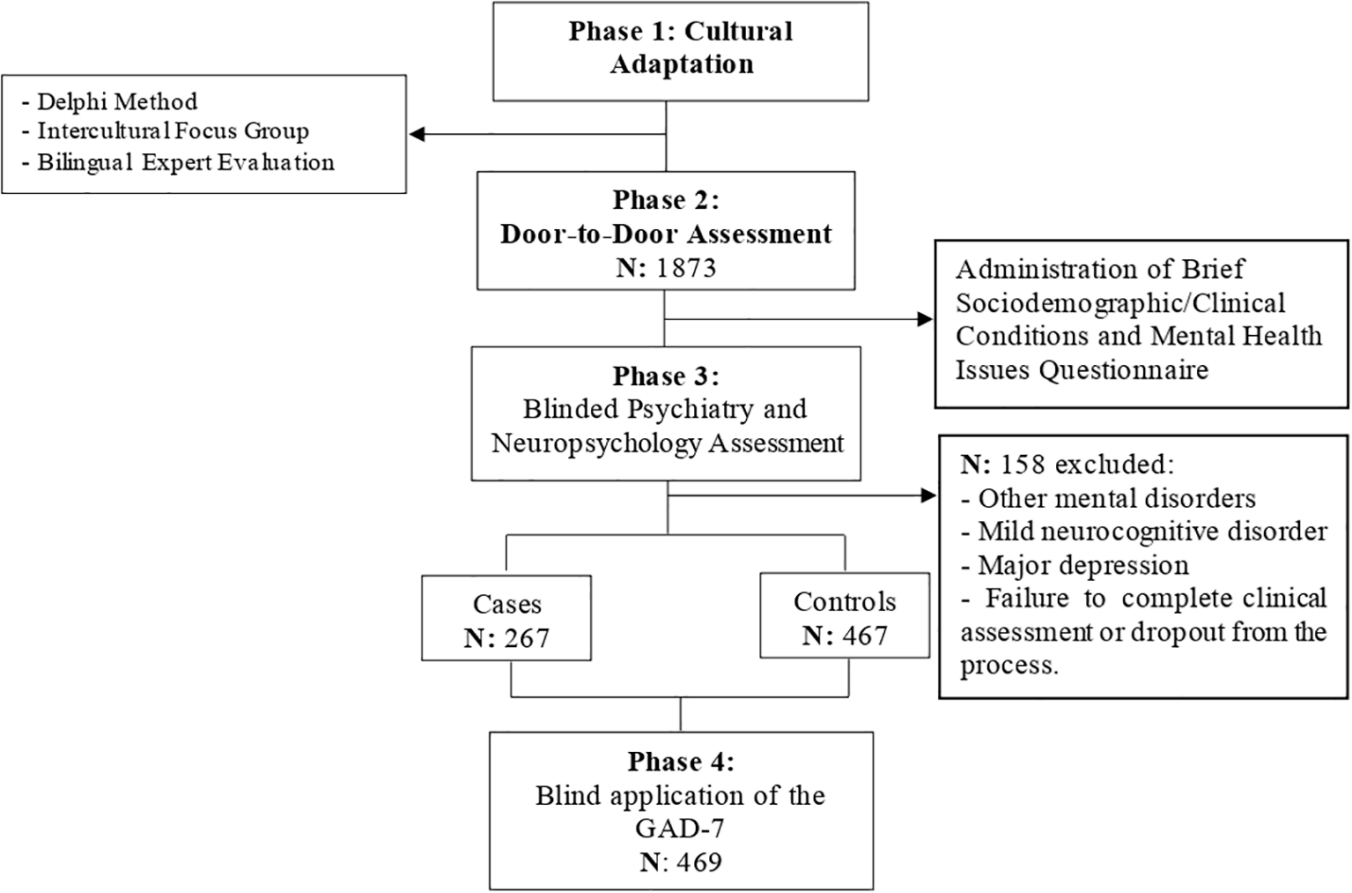
Study flowchart.

The study was conducted in four stages (**Figure 1**: Study flowchart). First, a linguistic adaptation of the GAD-7 items into Quechua was carried out using the Delphi method as a strategy to achieve community consensus. This process was developed by two members of the research team (IQR and CQA), together with a panel composed of eight bilingual community members, native Quechua speakers fluent in Spanish. The first round consisted of the individual review of the preliminary item translation, analyzing semantic clarity, conceptual equivalence, and cultural appropriateness. A second round was then held as an in-person group discussion (focus group), in which particularly sensitive terms—such as “anxiety” and “nervousness”—were collectively explored, and culturally relevant linguistic adjustments were proposed. This stage enabled consensus on the final wording of the items, respecting the Indigenous worldview, the principles of functional equivalence, and the criteria of community acceptability and comprehension.

Then, using the Quechua version of the GAD-7, the content of the test items was analyzed based on the judgment of four experts—specialists in psychiatry and mental health—with cultural and linguistic ties to Quechua. The experts provided valuable suggestions that contributed to improving the adaptation of specific terms and the response options of the GAD-7. Additionally, using the Typeform platform, and based on a specification table, the semantic definition and construct components of each item on the scale were analyzed. The judges followed the 5 criteria of Osterlind to complete the content validity (63).

Secondly, a door-to-door invitation was carried out targeting middle-aged adults from rural areas of the Peruvian Andes (Ayacucho, Apurímac, and Huancavelica); identified as Quechua Indigenous individuals, native Quechua speakers and bilingual in Spanish, with the aim of evaluating the effects of the post-COVID-19 pandemic on the mental health of this population. A total of 1,873 individuals responded to a brief initial questionnaire, which included 3 demographic questions (sex, age, and years of formal education); 3 cultural questions (whether Quechua was their mother tongue, whether they identified as part of the indigenous community, and whether they engaged in associated cultural practices); and a brief checklist on generalized anxiety symptoms, along with 2 clinical questions (symptoms of mood disorders before, during, and after the pandemic, and what illnesses or disorders they had).

Third, 890 of the 1,873 respondents underwent a psychiatric and neuropsychological evaluation using medical criteria and specialized scales to assess mood and neurocognitive disorders. Specialists did not have access to the door-to-door survey information. Based on the interdisciplinary evaluation and consensus, participants were assigned to two diagnostic groups (cases: 265 and healthy controls: 467). A total of 158 participants were excluded due to the presence of other conditions concurrent with GAD, findings of mild neurocognitive disorder, neuropsychiatric disorder, major depression, failure to complete the clinical evaluation or dropout.

Finally, the selected subjects (n=732) were referred to psychologists with mental health training who administered the GAD-7 in a blinded fashion. A total of 263 individuals did not attend the second appointment, resulting in a final sample of 469 assessed patients. Upon completing the assessment, the medical team met with the scientific team to analyze the results.

### 2.2 Instrument

The GAD-7 is a widely used scale for assessing Generalized Anxiety Disorder over the past two weeks, according to the DSM-5 (64). It contains 7 items, each scored on a 4-point Likert scale indicating the frequency of symptoms, ranging from 0 (not at all) to 3 (almost every day). The total GAD-7 score can range from 0 to 21, with a score of ≥10 indicating generalized anxiety disorder. The original study reported adequate sensitivity (0.92) and specificity (0.83) (58). Since then, good psychometric properties of the instrument have been reported worldwide (56, 65), and it is widely used in various mental health contexts (66, 67).

Based on the adapted version of the GAD-7 for the Spanish-speaking Peruvian population (68), we made the linguistic and cultural adaptations for the Quechua population (**Supplementary Data**: Quechua GAD-7 Test). In addition, a training program was designed for the field team that conducted the door-to-door surveys. This training included theoretical and practical sessions aimed at strengthening communication and interviewing skills and using the virtual platform for data collection. In addition, the field staff and the professionals conducting the blind evaluations were trained in mental health, emotional support techniques, and intercultural clinical assessment.

### 2.3 Data analysis

Content and linguistic validity were obtained using Aiken’s V confidence interval for each item of the instrument, using the Icaiken software (69). This confidence interval makes it possible to check whether the magnitude of the coefficient obtained is more significant than a predetermined minimally acceptable level (70–72). Its value ranges from 0 to 1, with a value of 1 indicating perfect agreement between the judges regarding the maximum validity score of the content evaluated.

Next, an analysis of the psychometric properties of the GAD-7 was conducted for the rural Quechua population of Peru. An exploratory factor analysis (EFA) was performed according to the KMO and sphericity criteria using the non-orthogonal oblimin method. For the confirmatory factor analysis (CFA), the weighted least squares mean, and variance adjusted (WLSMV) method was used. The following metrics were used for goodness-of-fit criteria: root mean square error of approximation (RMSEA) with an acceptable fit value, goodness-of-fit index (GFI), and standardized root mean square residual (SRMR). Reliability was then analyzed using the omega index, Cronbach’s alpha coefficient, and item-total correlation based on the final items of the instrument.

Finally, a diagnostic utility analysis was performed by comparing the performance of the GAD-7 with expert medical criteria using the aROC curve methodology. Due to the cultural characteristics of the population studied, the cutoff score suggested in the literature (≥10 points) was not selected to detect GAD symptoms. Instead, the cutoff score was chosen where the sensitivity (S) and specificity (S) values of the GAD-7 test were best balanced. Statistical analyses were performed using the R program, version 1.3.1056.

### 2.4 Ethical considerations

Informed consent was obtained from the study participants, who were informed about the objective of the research. All procedures were followed to comply with national and international ethical standards related to human research under the 1975 Helsinki Declaration, revised in 2008, for those subjects whose results indicated mood disorders, psychotherapy, and comprehensive clinical care were offered. This research is part of an international study aimed at analyzing mental health in the adult and elderly populations of Latin America and the Caribbean. The protocol was approved by the Ethics Committee of Universidad Nacional Autónoma Altoandina de Tarma, Perú (Ref. 01-2024)

## 3 Results

**Table 1** presents the content validity analysis of the GAD-7 items adapted for the Quechua population of Peru. All 7 items of the instrument showed adequate content validity (Aiken’s V), above 50%; making significant adjustments to the item statements unnecessary. Additionally, 6 out of 7 items demonstrated excellent inter-judge consensus, except for item 6, which was still very good, with over 75% global consensus.

**Table 1:** Evaluation of the Quechua GAD-7 items.

Items GAD	Osterlin Criterion	Judges				ME	Aiken	% Consensus	Confidence Interval	
		1	2	3	4				L Upper	L Lower
**1**	Rep	4	4	4	4	4	1	0,89%	.70	.92
	Pert	4	2	4	4	3,5	0,87			
	Comp	4	4	4	3	3,8	0,93			
	Inter	4	4	4	3	3,8	0,93			
	Clar	4	3	3	4	2,5	0,72			
**2**	Rep	4	4	4	4	4	1	0,91%	.72	.94
	Pert	4	4	4	3	3,8	0,93			
	Comp	4	4	4	4	4	1			
	Inter	4	3	4	3	3.5	0,87			
	Clar	4	2	4	3	3,2	0,75			
**3**	Rep	4	3	4	4	4	1	0,93%	.85	.95
	Pert	4	4	3	4	3,8	0,93			
	Comp	4	3	4	4	4	0,93			
	Inter	4	4	3	4	3,8	0,93			
	Clar	2	4	4	4	3,5	0,87			
**4**	Rep	4	4	4	4	4	1	0,98%	.91	.99
	Pert	4	4	4	4	4	1			
	Comp	4	4	4	4	4	1			
	Inter	4	4	4	3	3,8	0,93			
	Clar	4	4	4	4	4	1			
**5**	Rep	4	4	4	4	4	1	0,89%	.79	.92
	Pert	4	4	3	3	3,5	0,87			
	Comp	3	4	3	3	3,3	0,82			
	Inter	4	3	3	3	3,3	0,82			
	Clar	4	4	4	3	3,8	0,95			
**6**	Rep	3	4	3	4	3,5	0,7	0,76%	.65	.79
	Pert	3	4	4	3	3,8	0,76			
	Comp	4	4	4	4	4	1			
	Inter	3	3	3	4	3,3	0,66			
	Clar	3	3	4	3	3,3	0,66			
**7**	Rep	4	4	3	3	3,5	0,7	0,80%	.74	.84
	Pert	3	4	3	2	3	0,6			
	Comp	4	4	4	4	1	1			
	Inter	3	3	4	4	3,5	0,7			
	Clar	4	4	4	4	4	1			

Rep, Representativity; Pert, Pertinence; Comp, Comprehension; Inter, Interpretation; Clar, Clarity; ME, Mean; Lower. P, Lower Limit; Upper. L, Upper Limit.

In **Table 2**, the descriptive statistics and exploratory factor analyses of the items from the GAD-7 test are shown. The results of the EFA revealed a unidimensional structure that explained 66.11% of the data variance. Upon reviewing the factor loadings, it was determined that all 7 items of the test met the criteria to be part of the unidimensional structure (KMO = .900; p <.000). The factor loadings for the items exceeded.720, adhering to the recommendations of (67).

**Table 2:** Descriptive statistics and exploratory factor analysis of the Quechua GAD-7.

Items	ME	DS	kurtosis	Skewness	Confidence Intervals		Factor 1	Communality
					Lower Limit	Upper Limit		
1	1,29	0,92	-0,79	0,23	1,22	1,35	0,85	0,73
2	0,99	0,88	-0,23	0,67	0,92	1,05	0,88	0,78
3	1,28	0,96	-1,04	0,10	1,21	1,36	0,89	0,79
4	1,46	0,98	-1,02	0,07	1,38	1,53	0,89	0,79
5	0,95	0,86	-0,34	0,60	0,89	1,01	0,84	0,71
6	1,25	0,94	-0,72	0,37	1,18	1,33	0,71	0,50
7	1,18	1,05	-1,00	0,45	1,10	1,26	0,76	0,58

ME, Mean; DS, Standard Deviation.

A confirmatory factor analysis was conducted. The global fit indices indicated an adequate model fit (RMSEA = .059; GFI = .996; AGFI = .989; SRMR = .032). The incremental indices also met the established criteria (NFI = .996; RFI = .994), with values ≥.950 as recommended (70). The parsimony indices were acceptable (PNFI = .664; PGFI = .532), exceeding the standard criterion of ≥.500. **Table 3** presents the factor loadings and item communalities based on the analysis performed.

**Table 3:** Exploratory factor analysis.

In the past 15 days, have you felt or experienced:	Factor 1	Communality
1. A feeling of nervousness, anxiety, or being on edge?	0,85	0,73
2. Inability to avoid or control worrying?	0,88	0,78
3. Excessive worry about different things or situations?	0,89	0,79
4. Difficulty relaxing?	0,89	0,79
5. Restlessness to the extent that it is hard to stay still?	0,84	0,71
6. Easily annoyed or irritable?	0,71	0,50
7. Fear, as if something awful might happen?	0,76	0,58

**Figure 2:**
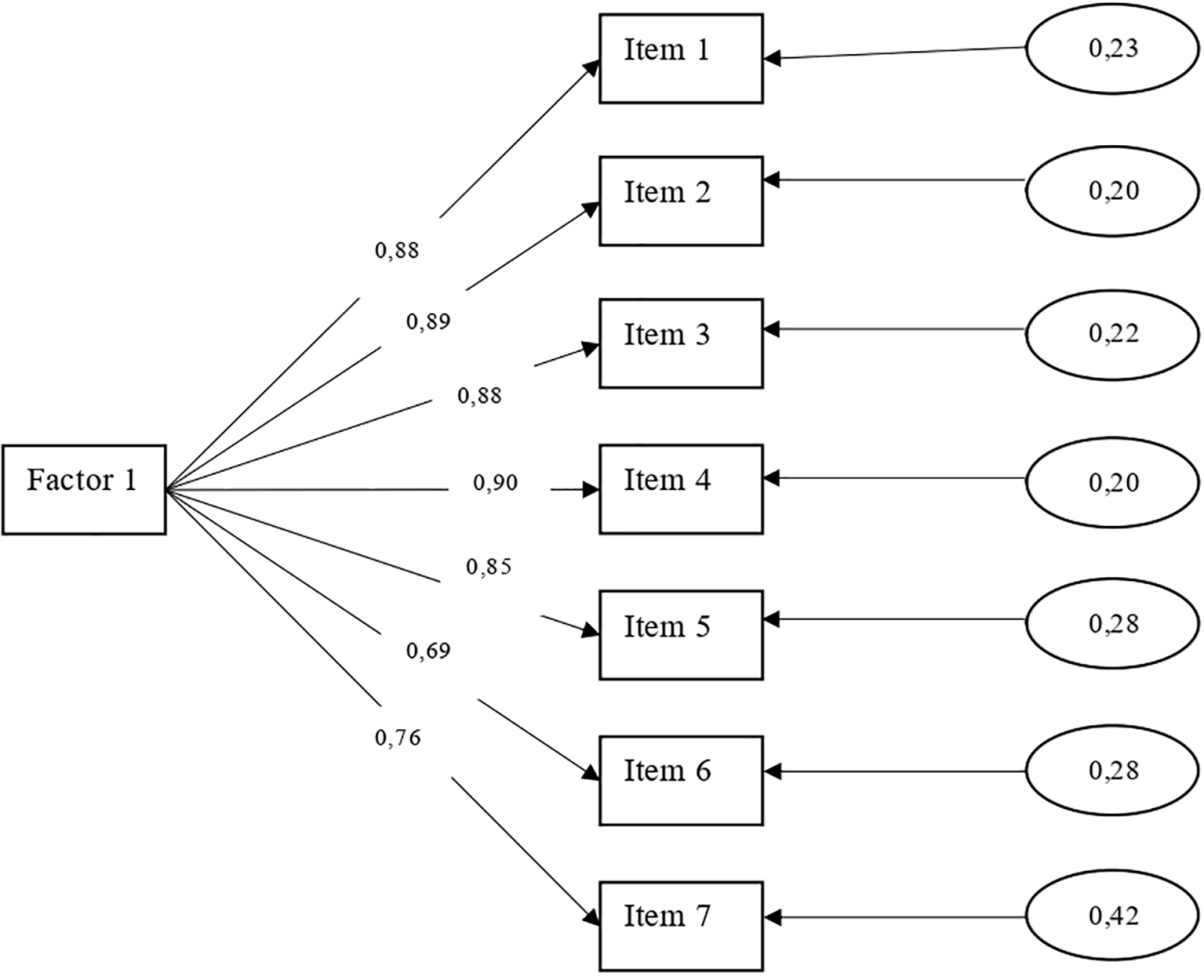
Unidimensional characteristic of the GAD-7 Test.

**Figure 2** shows the factor loadings for the 7 items on their respective scales. The factor has loadings ranging from.70 to.90. Finally, the correlation between the factors is low, which does not affect the discriminant validity of the instrument for detecting the theoretical construct (generalized anxiety) in the Quechua population of Peru.

Additionally, the 7 items of the GAD-7 were subjected to an internal consistency analysis to determine the reliability index of the scale. The Cronbach’s Alpha coefficient (α: 0.912) and the Omega index (ω: 0.85) were used for this. These results showed high reliability of the scale.

**Figure 3:**
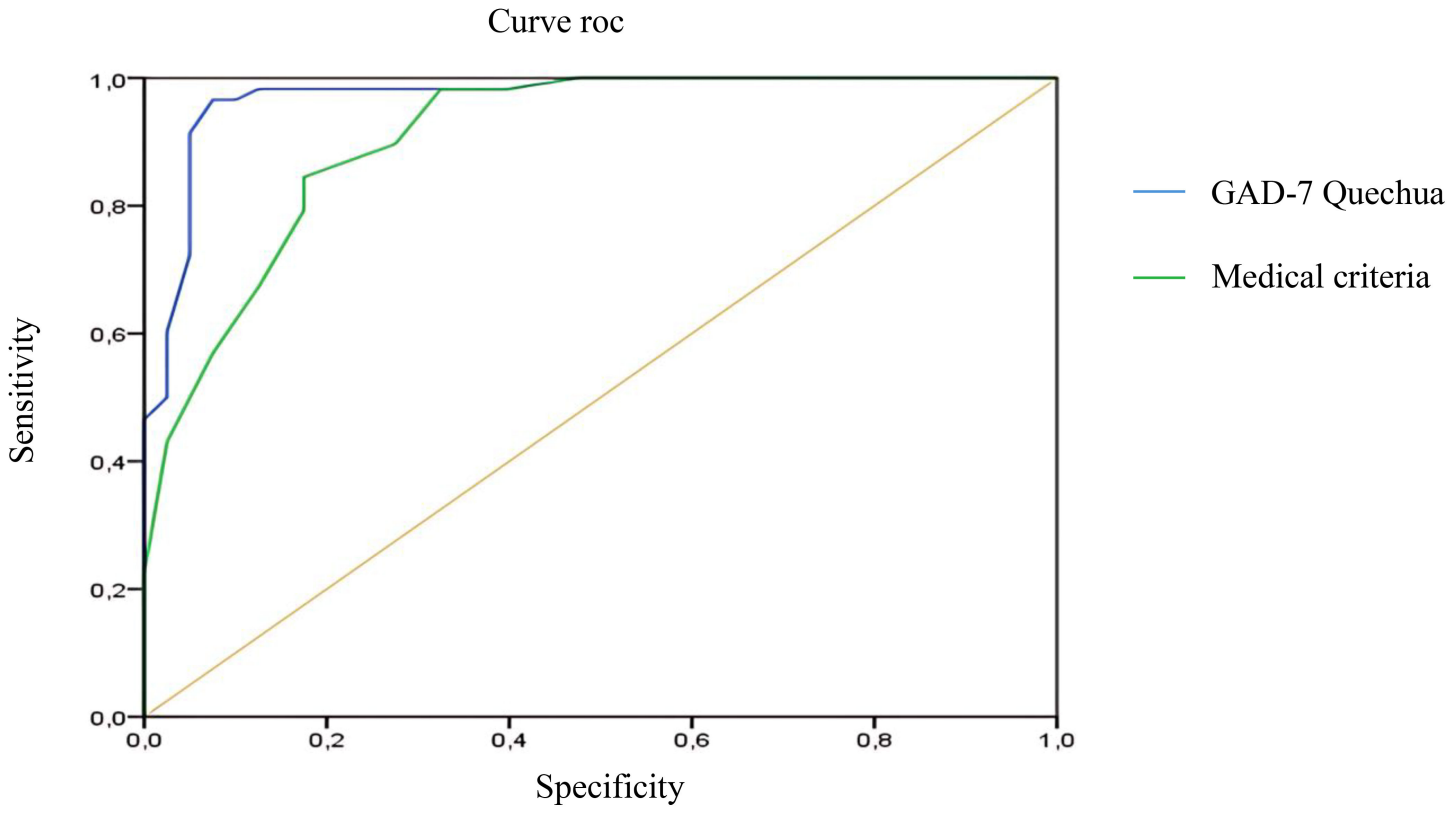
Compared ROC curve.

Finally, ROC curve analysis was conducted to compare the diagnostic utility of the Quechua version of the GAD-7 with the expert psychiatric medical diagnosis (**Figure 3**). The area under the curve for the medical criterion was high (aROC:.90), with the test slightly higher (aROC:.93). To establish the optimal cut-off point, the Youden index was used, selecting the value that simultaneously maximized sensitivity and specificity—that is, the point at which both parameters showed the best balance. With a cutoff score of ≥11 points, the GAD-7 exhibited Sensitivity (91.3) and Specificity (86.1) values higher than those shown by the medical criterion (S: 85.5; E: 80.6) for identifying generalized anxiety symptoms in general.

## 4 Discussion

We analyzed the diagnostic utility of the GAD-7 in Quechua-speaking populations of the Peruvian highlands, addressing its cultural adaptation, psychometric analysis, and diagnostic accuracy against the expert clinical criterion, using the area under the curve method.

In linguistic and cultural terms, the test the test showed adequate content values (Aiken’s V), with more than 50% consensus among the bilingual expert judges who reviewed the 7 items of the Quechua GAD-7. Thus, few linguistic adjustments were necessary, except for item 1, where the words “anxiety” and “nervousness” were problematic. These terms are rare or have no linguistic equivalents in Quechua as they are spoken in the highlands. It is important to note that Quechua does not have precise terms for concepts such as ‘fear’ and ‘nervousness’; emotions in this language are expressed through situational and bodily descriptions rather than abstract terms. According to Mannheim and Salas Carreño (73, 74), Quechua terms for emotions are deeply tied to bodily experience and social relationships, complicating the direct translation of Western psychological concepts such as ‘anxiety.’ Similarly, previous studies by Lévi-Strauss (75), showed that Indigenous languages tend to describe emotional states through concrete and situational references, reflecting a worldview in which emotions are linked to the environment and community relationships. In addition, Quechua is characterized as an agglutinative action language, where a single word has the potential to signify and express different emotions.

Thus, two perspectives come into conflict when assessing emotional states in Indigenous communities. The Western perspective, used when the goal is to ensure that participants understand precisely what is being measured, follows Western mental health terminology as recently described (76). This study used the terms “phutisk’alla/ansiedad nisqhawan.” This choice combines a Quechua term (“phutisk’alla”) with an additional explanation in Spanish (“ansiedad nisqhawan”). This approach is used to ensure that the concept of anxiety is adequately understood, as there may not be an exact equivalent in Quechua that fully captures the notion of “anxiety” as it is understood in Western psychiatry.

On the other hand, the term “ancha mancharisqha” (nervousness) also uses terms adapted or translated from Spanish into Quechua. In this case, the expression “mancharisqa” implies a reaction of alarm or fear, which is how nervousness could be understood in these contexts. This expression would be more easily understood and accepted by the target population (76).

In contrast, our study’s anthropological or community-based perspective focuses on respecting the indigenous worldview by using terms that correspond to emotions as they are understood in Quechua communities. In this sense, we used the term “llaquiska” (fear), which refers to intense worry or sadness, a relevant emotional concept in the Indigenous worldview. We also used the term “manchakusqachu” (nervousness), which refers to being frightened or alarmed. This aligns well with the concept of nervousness, especially when nervousness is perceived as a response to a perceived threat.

The Cjuno et al. (76), version of the GAD-7 combines Quechua and Spanish to ensure that clinical concepts are fully understood, which is helpful for accurate diagnosis within a medical-psychological framework. This approach is appropriate for use with Indigenous individuals living in either community or urban settings. In contrast, our version maintains greater fidelity to the language and Indigenous perspective, which may be more appropriate for rural Indigenous communities. However, it requires more context or explanation during the assessment (especially for item 1) to fully capture clinical nuances.

Moreover, Indigenous communities understand health, illness, and well-being differently from the Western perspective, integrating personal well-being with community, the land, and oral traditions (77–80). This makes it difficult to incorporate these perspectives into the individualistic model of Western health (81–83). Some clinical conceptualizations may lose meaning if they lack linguistic equivalents or intragroup acceptance from an Indigenous perspective (84–86). Furthermore, terms such as “distress,” “anxiety,” “nervousness,” or “depression” are either not used or are interpreted differently in these communities (85–87).

Therefore, to reduce cultural bias in the measurement of mental health issues, it is crucial to conduct cross-cultural adaptations with the active participation of Indigenous peoples, paying particular attention to integrating the holistic intercultural perspective of well-being from these communities (88, 89) as was done in our study and is reflected in the available evidence (90, 91). This may involve adapting or removing items from a test (52, 92–94); or, in some cases, creating new items (95, 96). This approach makes the standard Western test more culturally appropriate and usable in Indigenous contexts to assess mode (97–99); as described in a recent meta-analysis that analyzed the extent of tests used to screen for depression and anxiety in Indigenous adults worldwide (100). However, from an anthropological perspective on health, especially from an ethnological epistemology, it would be most appropriate for Indigenous communities to define mental health criteria and conduct measurements, analyses, interpretations, and decisions based on their cultural frameworks (101–104).

After cultural adaptation, we performed exploratory factor analysis (EFA) and confirmatory factor analysis (CFA) on the Quechua version of the GAD-7 used in our study. The EFA revealed a unidimensional structure that explained 66.11% of the variance in the data, with factor loadings ranging from 0.71 to 0.89, as recently reported in a study of a Quechua Collao-speaking population (76); this is a variant of Quechua. In this study, sedimentation or parallel analysis showed the presence of a single latent factor; similarly, KMO analyses (0.88, p = 0.01) confirmed these results, considering the linguistic and cross-cultural adaptations built into the test, showing adequate factor weights (0.64 - 0.85) (76).

Although there are very few studies using the GAD-7 or analyzing its psychometric properties in Indigenous populations, our results are consistent with the limited evidence available (76). In this context, a recent global meta-analysis shows that studies of anxiety and depression in indigenous populations have focused more on analyzing depression symptoms than anxiety, often using the PHQ-9 or shorter versions of it (100). In other cases, some studies have equated generalized anxiety disorder (GAD) with depression for analysis in Indigenous communities (86, 100). Exceptions include two studies that examined mental health indicators, including GAD, among Aboriginal people in Alberta, Canada (105) and South Dakota, USA (106), using a 2-item version of the GAD.

Nevertheless, the findings of unidimensionality in our analyses are consistent with previous studies that have validated the GAD-7 in culturally diverse societies, such as Japanese (107), Korean (108), Chinese (109, 110) and African (43, 111) populations. In addition, the GAD-7 is invariant concerning gender, level of education, and membership in culturally diverse communities (54, 112–114); suggesting that these variables do not influence test scores (62, 115). Similarly, these psychometric indicators have been confirmed in various Western populations (49, 51, 56, 58, 116); providing robust evidence for the test’s unidimensionality.

On the other hand, the CFA showed adequate fit indices, with an RMSEA of 0.100, a CFI of 0.996, and an SRMR of 0.056, supporting the robustness of the factorial structure in the Quechua Peruvian population. These fit indices are comparable to those obtained in a recent study with Indigenous populations (76) and in global multicultural contexts, such as in China (110) and Russia (117).

In addition, reliability analyses of the GAD-7 showed optimal values for Cronbach’s alpha (α: 0.912) and omega (Ω: 0.85). This is consistent with similar findings in multicultural populations worldwide (37, 45, 50, 52, 104, 106, 110, 114–119, 128–131), where internal consistency values (α: between 0.82 - 0.94; Ω: between 0.894 - 0.90) like ours have been reported, although most studies were conducted in culturally and linguistically diverse populations, with only one focusing on indigenous peoples (76). These results suggest that the 7 items of the GAD-7 are consistent and correlate with each other, ensuring the integrity of the test to accurately measure the construct despite the cultural and racial differences in which it has been tested.

Finally, we analyzed the diagnostic utility of the GAD-7 by comparing it to the psychiatric expert criterion. In this regard, the Quechua version of the GAD-7 (aROC:.93) showed greater diagnostic accuracy than the psychiatric evaluation (aROC:.90) for detecting symptoms of generalized anxiety disorder (GAD). Recently, our research group (120), conducted a phase 1 case-control study, reporting high diagnostic utility of the GAD-7 (aROC: 0.91) in a clinical Quechua Indigenous sample. Apart from these two studies, there are no similar studies in Indigenous populations in the current literature, although there are some in rural communities speaking Latvian and Russian (62) and in China (121); where AUC values were reported to range between 0.76, 0.86, and 0.97, respectively. These indicators reflect the clinical benefit of using the GAD-7 in diverse and geographically remote settings. Additionally, it is worth noting the existence of a 2-item version of the test, which has been analyzed in rural areas (122, 123); showing AUC ranges varying between 0.69 and 0.954.

Moreover, by balancing sensitivity and specificity values, our study selected a cut-off score of 11 points to identify GAD symptoms. With one point more than in the original study (49), a sensitivity of 90.3% and specificity of 84.1% were achieved-both higher than those reported using the medical criterion (S: 85.5%; E: 80.6%). This cut-off point of ≥11 had already been proposed in a previous study conducted by our team, in which we reported sensitivity (92.56%) and specificity (89.35%) values of comparable magnitude (120), as this threshold offered a better balance between sensitivity and specificity in our sample, optimizing diagnostic performance according to the Youden Index. where we reported similar sensitivity (92.56%) and specificity (89.35%) values. Taken together, these results strengthen the evidence supporting the cross-cultural applicability of the GAD-7 as an effective screening tool in Indigenous populations.

However, we are struck by the diversity of cut-off scores (5–10) and wide ranges of sensitivity (56-94%) and specificity (46-94.4%) available in the literature. In this regard, Mughal et al. (124), conducted a systematic review of GAD screening tools in low- and middle-income countries and confirmed this variability. They reported that the GAD-7 was one of the most widely used and accurate diagnostic tests for detecting anxiety disorders; however, it showed a wide range of sensitivity (57-94%) and specificity (53-94%). This variability was associated with the region in which it was administered, participant characteristics, and methodological aspects.

An example of these variations in AUC values for the GAD-7 is the study by Vrublevska et al. (62); who validated the GAD-7 in urban and rural primary care settings in Latvia, a country with cultural and linguistic ties to Russia. In this study, two versions of the GAD-7 were analyzed. The cut-off score (≥5) and the sensitivity (0.70) and specificity (0.79) values for the Latvian version differed from those obtained for the Russian version (cut-off ≥7; S: 73.3 and E: 84.1). These findings highlight the importance of adapting GAD-7 cutoff points to specific populations and show that the optimal score may vary significantly between different cultural and linguistic groups. They also confirm the clinical utility of the GAD-7 as a tool for the detection of generalized anxiety disorder in multicultural contexts.

Despite these findings, our study has several limitations that should be considered. First, probabilistic sampling of participants was not used, which may limit the generalizability of the findings to other indigenous populations in the country. However, a rigorous double-blind selection and assessment process was used, which is rarely seen in similar studies and enhances the quality of the analyses. Second, although a thorough cultural adaptation of the GAD-7 was carried out, it is possible that some linguistic and cultural nuances were not fully captured, especially since only one variant of Quechua was considered. In addition, it has been challenging to reconcile the indigenous perspective on mental health with the Western perspective. Therefore, we believe continuing with studies that convey these cultural specificities is necessary. Finally, a gold standard test could not be used for convergent analysis with the GAD-7 due to its non-existence. These limitations suggest the need for future studies that address these aspects, as such studies are very scarce worldwide.

Considering these points, we can conclude that the Quechua version of the GAD-7 is appropriate, reliable, and effective in identifying mood variation in rural indigenous communities in Peru and overcoming cultural barriers. It has adequate clinical utility indicators for the detection of generalized anxiety disorder symptoms. This is the first study in the world to compare the accuracy of the GAD-7 test with expert medical criteria for detecting GAD in a rural indigenous population. This is excellent news for community health settings, especially in remote areas of a country where access to medical services is often limited. Therefore, having a quick to administer, easy to score and interpret clinically and culturally validated test is an excellent strategy for early and transcultural detection of mental health problems in multicultural settings (125–127). Additionally, it helps increase health coverage indicators in rural and indigenous contexts within the country.

## Data Availability

The raw data supporting the conclusions of this article will be made available by the authors, without undue reservation.

## Supplementary material

Supplementary Data S1 GAD-7 test in Quechua.
