# Supplementary material for: Clinical utility of the GAD-7 for detecting generalized anxiety in Quechua indigenous people
Source: Front Psychiatry. 2025 May 30;16:1565895. doi: 10.3389/fpsyt.2025.1565895 (PMC12163617; doi:10.3389/fpsyt.2025.1565895)
Supplement: Supplementary Data S1 — GAD-7 test in Quechua. [file DataSheet1.docx]

**Supplementary data: S1. GAD-7 test in Quechua**

| **¿Kay chunka kimsayuq siqi:** |
| --- |
| 1. Manchakusqachu, llakisqa utaq ancha piñasqachu kachkanki? |
| 1. Manachu llakikita qunqayta atirqanki |
| 1. Huk niraq  hamutaykunata llumpaytachu llakichisunki |
| 1. Sasachu hampaq hawka kayniki |
| 1. Ancha mana allintachu qasilla kayta atinki, hinaspa mana qasilla kayta atinkichu. |
| 1. Manachu sasachakunki piñakuyta, llakikuyta utqayman |
| 1. Imapas mana allin hina manchakuymi kawan |
