# Supplementary material for: Clinical utility of the GAD-7 for detecting generalized anxiety in Quechua indigenous people
Source: Front Psychiatry. 2025 May 30;16:1565895. doi: 10.3389/fpsyt.2025.1565895 (PMC12163617; doi:10.3389/fpsyt.2025.1565895)
Supplement: Supplementary file 2 [file DataSheet2.docx]

**Supplementary data: S1. GAD-7 test in Quechua (English Copy)**

| **In the past 15 days, have you felt or experienced:** |
| --- |
| 1. A feeling of nervousness, anxiety, or being on edge? |
| 1. Inability to avoid or control worrying? |
| 1. Excessive worry about different things or situations? |
| 1. Difficulty relaxing? |
| 1. Restlessness to the extent that it is hard to stay still? |
| 1. Easily annoyed or irritable? |
| 1. Fear, as if something awful might happen? |
